# Association of the triglyceride and glucose index with low muscle mass: KNHANES 2008–2011

**DOI:** 10.1038/s41598-020-80305-1

**Published:** 2021-01-11

**Authors:** Jung A. Kim, Soon Young Hwang, Ji Hee Yu, Eun Roh, So-hyeon Hong, You-Bin Lee, Nam Hoon Kim, Hye Jin Yoo, Ji A. Seo, Nan Hee Kim, Sin Gon Kim, Sei Hyun Baik, Kyung Mook Choi

**Affiliations:** 1 Division of Endocrinology and Metabolism, Department of Internal Medicine, College of Medicine, Korea University, Seoul, Republic of Korea; 2 Department of Biostatistics, Korea University College of Medicine, Seoul, Republic of Korea

**Keywords:** Biomarkers, Endocrinology

## Abstract

The triglyceride-glucose (TyG) index is a simple surrogate marker of insulin resistance. We evaluated the association of the TyG index with low muscle mass using a nationwide population-based representative data. This is a cross-sectional study that included 9477 participants aged ≥ 40 years from the Korea National Health and Nutrition Examination Survey between 2008 and 2011. The TyG index was calculated as ln[triglyceride (mg/dL) × fasting plasma glucose (mg/dL)/2]. Dual-energy X-ray absorptiometry was used to measure appendicular lean mass (ALM). Low muscle mass was defined an ALM/weight of 2 standard deviations (SD) below of young participants. The overall prevalence of low muscle mass was 4.7%. The prevalence of low muscle mass increased linearly with the quartiles of the TyG index, 2.5%, 4.2%, 5.6%, and 6.7% in Q1–Q4, respectively. The TyG index was negatively associated with ALM/weight both in men (*r* = − 0.302) and women (*r* = − 0.230). The odds ratio (OR) for low muscle mass was 2.08 in the highest quartile compared to the lowest quartile. High TyG index was associated with an increased risk of low muscle mass (OR for 1SD increase: 1.13). Increased TyG index was associated with the risk of low muscle mass independent of confounding factors.

## Introduction

Age related loss of muscle mass with decreased muscle strength and physical performance is called sarcopenia. From the age of 40 years, skeletal muscle decreases by 8% per decade until age 70 years and then, accelerates to 15% per decade and this associated with decreased muscle strength^1^. Sarcopenia is related to falls, hospitalization, frailty, morbidity, and mortality^2^. In addition, sarcopenia affects inflammatory response and whole-body metabolism^3^ and is related to cardiometabolic diseases^4–8^. With an aging society, the increased impact of sarcopenia has increased burden on individuals and national health-related policies. Therefore, it is important to recognize patients who are at risk of sarcopenia in advance. Skeletal muscle is a primary organ for insulin disposal. Insulin resistance is the main pathology of development of low muscle mass^9^; evaluating insulin resistance might be valuable to identify subjects with loss of muscle mass.

The triglyceride-glucose (TyG) index is calculated using fasting glucose and triglyceride levels (ln(triglyceride(mg/dL) × glucose (mg/dL)/2))^10^. The TyG index is a simple surrogate marker of insulin resistance, which is comparable with the hyperinsulinemic-euglycemic clamp test and homeostasis assessment of insulin resistance (HOMA-IR)^11,12^. Recently, the TyG index is being considered more predictive than HOMA-IR in assessing the risk of insulin resistance related metabolic diseases such as metabolic syndrome^13^, type 2 diabetes (T2DM)^14^, and non-alcoholic fatty liver disease (NAFLD)^15^. Increased insulin resistance can mediate decreased glucose uptake in the skeletal muscle and hepatic glucose usage that can lead to elevated plasma glucose and triglyceride levels. In addition, decreased muscle mass also induces insulin resistance^16^. Given the close relationship between insulin resistance and low muscle mass, the elevated TyG index may be a risk indicator for low muscle mass. However, there has been no previous study demonstrating the clinical significance of TyG index in implicating a role for low muscle mass.

In this study, we explored the association between the TyG index and the risk of low muscle mass using data from the Korea National Health and Nutrition Examination Survey (KNHANES).

## Methods

### Study population

**Figure 1:**
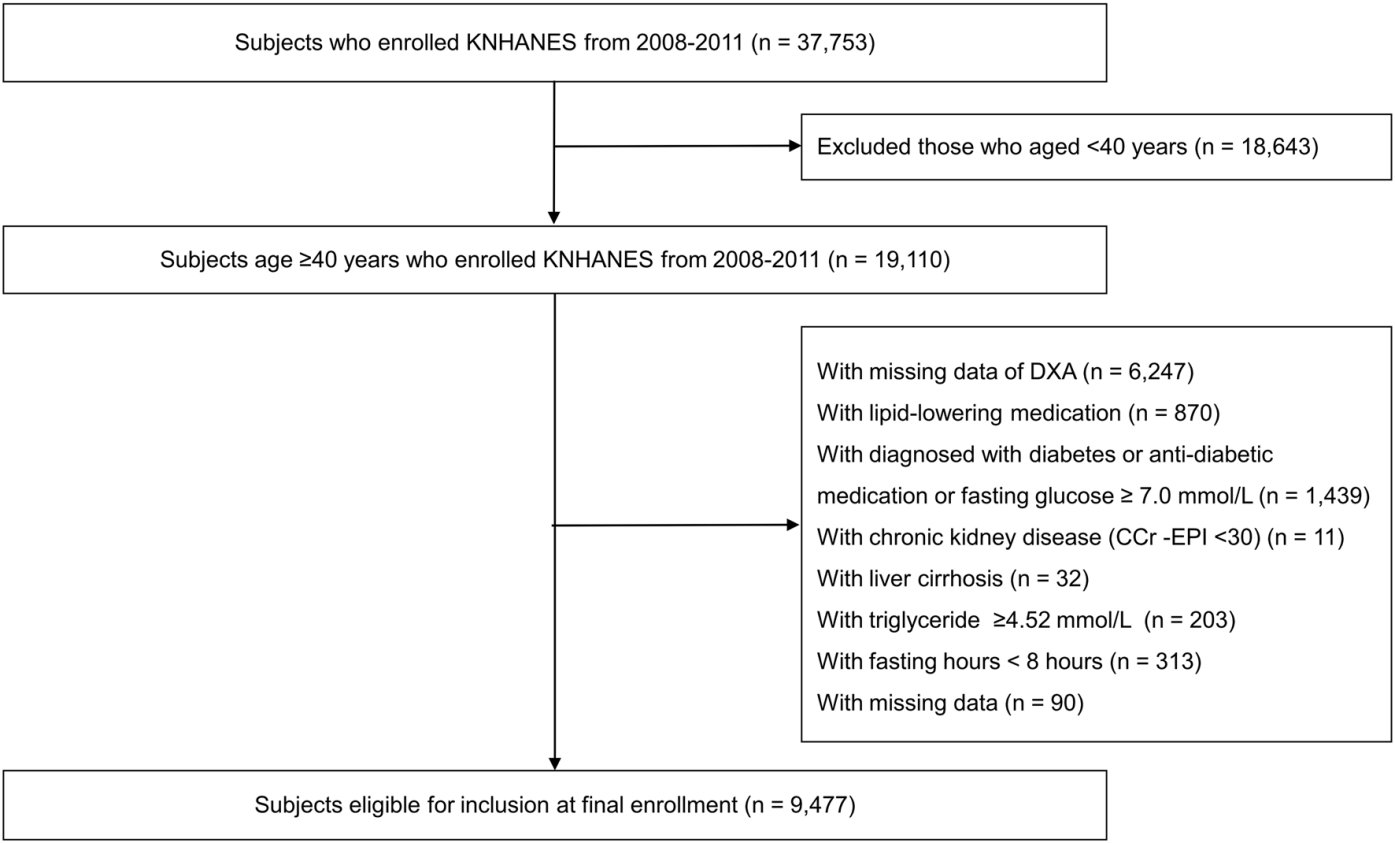
Flow chart of the study. DXA, Dual-energy X‐ray absorptiometry; KNHANES, Korea National Health and Nutrition Examination Survey; *TyG index* triglyceride-glucose index.

This study was conducted based on the data from the KNHANES IV-V of 2008–2011. The KNHANES is a nationwide, population-based survey to monitor the general health and nutrition status of the South Korean population. The KNHANES has been conducted annually since 1998, and data comprise anonymized personal information including medical history, anthropometric characteristics, lifestyle and dietary habits, and laboratory tests^17^. From the 37,753 participants, we excluded participants with younger than 40 years (n = 18,643), no data of whole body dual-energy X‐ray absorptiometry (DXA) (n = 6247), taking lipid-lowering medication (n = 870), diagnosed with diabetes or taking anti-hyperglycemic medication, or fasting glucose ≥ 7.0 mmol/L (n = 1439), chronic kidney disease (n = 11), liver cirrhosis (n = 32), triglyceride levels ≥ 4.52 mmol/L (n = 203), fasting time < 8 h (n = 313), and missing data (n = 90) (Fig. 1). In total, 9477 participants were eligible for the analysis. Our study protocol was approved by the Institutional Review Board (IRB) of Korea University and performed in accordance with the Declaration of Helsinki of the World Medical Association (2019GR0428). The requirement for written informed consent was waived by the IRB because anonymous and de-identified information provided by KNHANES was used for analysis. The data of the KNHANES are publicly available through the KNHANES website.

### Measurements and definitions

Venous samples were obtained after ≥ 8 h of overnight fasting and were transported to the central laboratory. The concentration of blood urea nitrogen (BUN), creatinine, aspartate transaminase (AST), alanine aminotransferase (ALT), total cholesterol (TC), triglyceride, high-density lipoprotein cholesterol (HDL-C), and glucose was measured enzymatically using the Hitachi automatic analyser 7600 (Tokyo, Japan). Low-density lipoprotein cholesterol (LDL-C) was calculated using the Friedewald formula equation^18^. For HDL-C, calibrated values were used to be comparable with the Center for Disease Control and Prevention (CDC) reference^19^. The TyG index was calculated as ln [triglyceride (mg/dL) x fasting plasma glucose (mg/dL)/2]^10^. Vitamin D was measured using the radioimmunoassay method with the 1470 wizard gamma-counter (PerkinElmer/Finland). All methods were carried out in accordance with relevant guidelines and regulations. Diabetes was defined as diagnosed with diabetes or taking anti-hyperglycemic medications or fasting glucose ≥ 7.0 mmol/L. Hypertension (HTN) was defined as diagnosed with HTN or systolic blood pressure (BP) ≥ 140 mmHg or diastolic BP ≥ 90 mmHg or taking anti-hypertensive medications. BP was manually measured three times, the mean of the last two records was used. A current smoker was defined as ≥ 5 packs of cigarettes ever. Heavy alcohol consumption was defined as ≥ 7 drinks/week in men and ≥ 5 drinks/week in women. Regular exercise was defined as a moderate-intensity exercise ≥ 30 min and ≥ 5 times per week or a high-intensity exercise ≥ 20 min at a time and ≥ 3 times per week. Body mass index (BMI) was calculated as weight (kg)/height in meters squared (m^2^).

### Definition of low muscle mass

DXA (QDR 4800A; Hologic, Bedford, MA) was used to evaluate body composition. Appendicular lean mass (ALM [kg]) was defined as the summation of the lean soft tissue mass of both arms and legs^20^. We used ALM adjusted by weight (ALM/weight) as the appendicular muscle mass index as adapted from the definition of Janssen et al.^21^. Low muscle mass was defined as ALM/weight < 2 standard deviation (SD) below the mean of a young reference group (age 20–39 years), with a cut-off of 28.8% for men and 22.8% for women.

### Statistical analyses

Data are expressed as mean ± SD and a number (percentage). Student’s t-tests and chi-square tests were used to compare the difference between groups. Age-adjusted Pearson’s partial correlation analysis was conducted to evaluate the correlations between the TyG index and metabolic variables, BMI, and lean muscle mass. Multivariate logistic regression analysis was used to calculate the odds ratio (OR) and 95% confidence interval (95% CI) for low muscle mass according to the quartiles of the TyG index after adjustment for age, sex, smoking status, heavy alcohol consumption, physical activity, systolic BP, TC, creatinine, AST, ALT, and vitamin D. The risk of low muscle mass with each 1-SD increment of TyG index was evaluated after adjustment for confounding factors. Statistical analysis was performed using SPSS software (version 20.0 for Windows; SPSS, Chicago, IL, the USA). A two-sided P*-*value of < 0.05 was considered statistically significant.

### Ethics approval and consent to participate

Our study protocol was approved by the Institutional Review Board (IRB) of Korea University and performed in accordance with the Declaration of Helsinki of the World Medical Association (2019GR0428). The requirement for written informed consent was waived by the IRB because anonymous and de-identified information provided by KNHANES was used for analysis.

## Results

### Baseline characteristics

**Figure 2:**
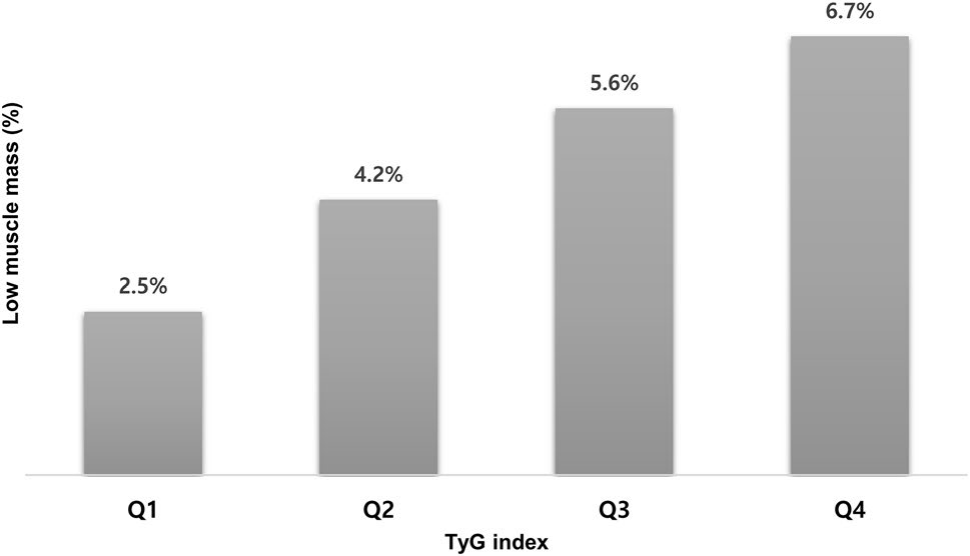
Prevalence of low muscle mass according to quartiles of the TyG index. *TyG index* triglyceride-glucose index.

Table 1 shows the baseline characteristics of participants according to the quartiles of the TyG index. Among the 9477 participants, the prevalence of low muscle mass was 4.7% (3.2% in men and 5.8% in women). Age, representation of men, WC, BMI, systolic BP, diastolic BP, glucose, TC, triglyceride, AST, ALT, vitamin D, the prevalence of low muscle mass, HTN, current smoker, and heavy alcohol consumption increased with the TyG index quartile increment. HDL-C, BUN, and regular exercise were higher in the lower quartiles of the TyG index than in the higher quartiles. The prevalence of low muscle mass increased from 2.5%, 4.2%, 5.6%, and 6.7% in the first, second, third, and fourth quartile of the TyG index, respectively (Fig. 2). Except for the prevalence of current smoker and heavy alcohol consumption, the anthropometric and metabolic risk factors were higher in low muscle mass group than normal group (Table 2). When we divided participants into men and women, WC, BMI, systolic BP, diastolic BP, glucose, TC, triglyceride, AST, ALT, TyG index, prevalence of HTN, current smoker, and heavy alcohol consumption increased parallel with increased quartiles of the TyG index both in men and women (Supplementary Table S1).

**Table 1:** Baseline characteristics according to the quartiles of the TyG index.

	TyG index				P-value
	Q1	Q2	Q3	Q4	
	n = 2365	n = 2377	n = 2366	n = 2369	
Men (%)	759 (32.1%)	899 (37.8%)	1054 (44.5%)	1286(54.3%)	< 0.001
Age (years)	54.4 ± 11.3	56.8 ± 11.5	57.7 ± 11.4	57.8 ± 11.2	< 0.001
Waist circumference (cm)	77.4 ± 8.5	80.6 ± 8.9	83.1 ± 8.5	86.1 ± 8.0	< 0.001
Body mass index (kg/m^2^)	22.5 ± 2.8	23.4 ± 3.0	24.1 ± 3.1	24.8 ± 2.8	< 0.001
Systolic blood pressure (mmHg)	117.6 ± 17.5	121.9 ± 17.3	124.1 ± 17.4	127.8 ± 17.2	< 0.001
Diastolic blood pressure (mmHg)	75.3 ± 10.3	77.3 ± 10.2	79.0 ± 10.4	81.4 ± 10.5	< 0.001
Fasting plasma glucose (mmol/L)	5.0 ± 0.4	5.2 ± 0.5	5.3 ± 0.5	5.5 ± 0.5	< 0.001
Total cholesterol (mmol/L)	4.7 ± 0.8	4.9 ± 0.9	5.1 ± 0.9	5.3 ± 0.9	< 0.001
Triglyceride (mmol/L)	0.7 ± 0.1	1.1 ± 0.1	1.5 ± 0.2	2.6 ± 0.7	< 0.001
HDL-C (mmol/L)	1.4 ± 0.3	1.3 ± 0.3	1.2 ± 0.3	1.1 ± 0.2	< 0.001
LDL-C (mmol/L)	3.0 ± 0.7	3.2 ± 0.8	3.3 ± 0.8	3.0 ± 0.9	< 0.001
Aspartate aminotransferase (IU/L)	21.6 ± 9.2	22.1 ± 9.5	22.8 ± 13.6	25.3 ± 14.2	< 0.001
Alanine aminotransferase (IU/L)	17.4 ± 10.6	19.1 ± 11.3	21.5 ± 21.6	25.6 ± 17.3	< 0.001
Blood urea nitrogen (mmol/L)	5.4 ± 1.5	5.4 ± 1.5	5.3 ± 1.5	5.2 ± 1.4	< 0.001
Creatinine (μmol/L)	70.7 ± 17.7	70.7 ± 17.7	70.7 ± 17.7	70.7 ± 17.7	< 0.001
Vitamin D (nmol/L)	18.9 ± 7.2	19.3 ± 7.2	19.3 ± 7.1	19.6 ± 7.1	0.024
TyG index	7.9 ± 0.2	8.4 ± 0.1	8.7 ± 0.1	9.3 ± 0.3	< 0.001
ALM/weight (%)	30.0 ± 4.5	29.6 ± 4.5	29.6 ± 4.6	29.8 ± 4.3	0.002
Low muscle mass 1SD (%)	397 (16.8%)	606 (25.5%)	727 (30.7%)	850 (35.9%)	< 0.001
Low muscle mass 2SD (%)	58 (2.5%)	100 (4.2%)	132 (5.6%)	158 (6.7%)	< 0.001
Hypertension (%)	572 (24.3%)	772 (32.6%)	975 (41.4%)	1188 (50.3%)	< 0.001
Current smoker (%)	303 (12.9%)	374 (15.8%)	467 (19.9%)	630 (26.7%)	< 0.001
Heavy alcohol consumption (%)	269 (11.5%)	325 (13.7%)	408 (17.3%)	609 (25.9%)	< 0.001
Regular exercise (%)	633 (27.0%)	617 (26.2%)	607 (25.8%)	537 (22.8%)	0.005

Data are expressed as mean ± SD or n (%).

*ALT* alanine aminotransferase; *ALM* Appendicular lean mass; *HDL-C* High-density lipoprotein cholesterol; *LDL-C* Low-density lipoprotein cholesterol; *SD* standard deviation; *TyG index* triglyceride-glucose index.

**Table 2:** Comparison between the normal and low muscle mass groups in men and women.

	Men			Women		
	Normal	Low muscle mass		Normal	Low muscle mass	
	n = 3870	n = 128	P-value	n = 5159	n = 320	P-value
Age (years)	56.8 ± 11.4	64.2 ± 11.1	< 0.001	56.1 ± 11.3	61.3 ± 10.9	< 0.001
Waist circumference (cm)	84.0 ± 8.2	94.2 ± 8.7	< 0.001	79.4 ± 8.7	89.1 ± 9.3	< 0.001
Body mass index (kg/m^2^)	23.6 ± 2.8	26.8 ± 3.0	< 0.001	23.5 ± 3.0	27.3 ± 3.5	< 0.001
Systolic blood pressure (mmHg)	124.2 ± 17.0	130.4 ± 16.2	< 0.001	121.4 ± 18.2	127.9 ± 17.3	< 0.001
Diastolic blood pressure (mmHg)	80.3 ± 10.7	81.4 ± 9.9	0.262	76.6 ± 10.2	78.7 ± 10.2	< 0.001
Fasting plasma glucose (mmol/L)	5.3 ± 0.5	5.5 ± 0.6	< 0.001	5.2 ± 0.5	5.3 ± 0.5	< 0.001
Total cholesterol (mmol/L)	4.9 ± 0.9	5.0 ± 1.1	0.125	5.1 ± 0.9	5.4 ± 1.0	< 0.001
Triglyceride (mmol/L)	1.6 ± 0.9	1.8 ± 0.8	< 0.019	1.3 ± 0.7	1.6 ± 0.8	< 0.001
HDL-C (mmol/L)	1.2 ± 0.3	1.1 ± 0.3	< 0.001	1.3 ± 0.3	1.3 ± 0.3	0.115
LDL-C (mmol/L)	3.0 ± 0.8	3.1 ± 1.0	0.068	3.2 ± 0.8	3.4 ± 0.9	< 0.001
Aspartate aminotransferase (IU/L)	25.3 ± 15.6	25.2 ± 8.4	0.962	21.2 ± 7.9	22.5 ± 9.7	< 0.001
Alanine aminotransferase (IU/L)	24.6 ± 20.2	26.3 ± 13.7	0.319	18.0 ± 11.4	21.6 ± 18.0	0.016
Blood urea nitrogen (mmol/L)	5.6 ± 1.5	5.7 ± 1.9	0.337	5.1 ± 1.4	5.5 ± 1.6	0.001
Creatinine (μmol/L)	79.6 ± 8.8	88.4 ± 17.7	0.004	61.9 ± 8.8	61.9 ± 8.8	0.047
Vitamin D (nmol/L)	21.2 ± 7.3	18.9 ± 6.7	< 0.001	18.0 ± 6.8	16.7 ± 6.2	0.001
TyG index	8.7 ± 0.6	8.8 ± 0.5	< 0.001	8.5 ± 0.5	8.7 ± 0.5	< 0.001
ALM/weight (%)	34.1 ± 2.6	27.6 ± 1.0	< 0.001	27.0 ± 2.4	21.7 ± 0.9	< 0.001
Hypertension (%)	1556 (40.4%)	84 (65.6%)	< 0.001	1688 (32.8%)	179 (56.1%)	< 0.001
Current smoker (%)	1495 (38.9%)	33 (25.8%)	0.003	235 (4.6%)	11 (3.5%)	0.353
Heavy alcohol consumption (%)	1267 (32.9%)	29 (23.0%)	0.019	288 (5.6%)	27 (8.5%)	0.032
Regular exercise (%)	1039 (27.0%)	27 (21.3%)	0.150	1201 (25.0%)	127 (19.9%)	0.202

Data are expressed as mean ± standard deviation or n (%).

*ALM* appendicular lean mass; *HDL-C* High-density lipoprotein cholesterol; *LDL-C* low-density lipoprotein cholesterol.

### Associations of the TyG index with clinical, metabolic, and anthropometric parameters

**Figure 3:**
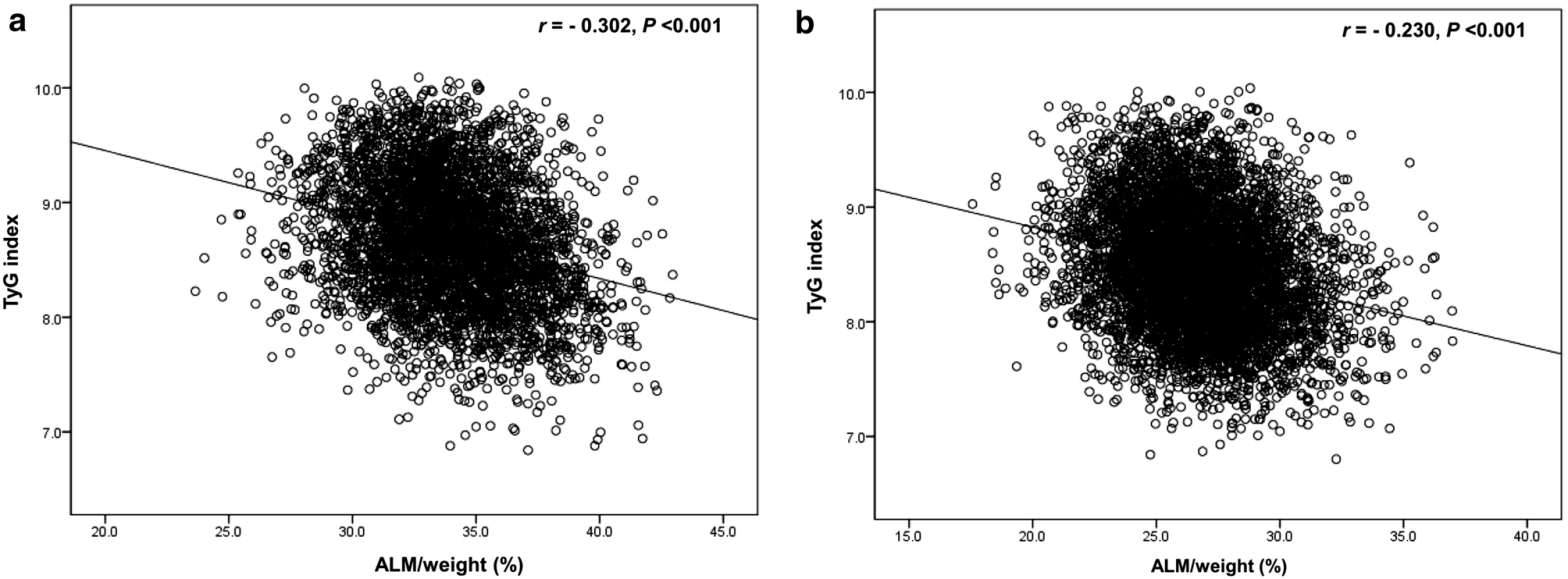
Scatter plot of the TyG index with ALM/weight in (**a**) men and (**b**) women. *ALM* appendicular lean mass; *TyG index* triglyceride-glucose index.

After adjustment for age, the TyG index was negatively associated with ALM/weight both in men (*r* = − 0.302, *p* < 0.001) and women (*r* = − 0.230, *p* < 0.001) (Table 3, Fig. 3). WC, BMI, glucose, systolic BP, diastolic BP, TC, triglyceride, creatinine, AST, and ALT were positively associated and TyG index, HDL-C, and vitamin D were negatively associated with the TyG index in both sexes. LDL-C was positively correlated with the TyG index in men and negatively correlated in women.

**Table 3:** Age-adjusted Spearman partial correlation analysis of the TyG index with clinical, metabolic, and body composition parameters.

	Men		Women	
	*r*	*P*	*r*	*P*
ALM/weight	− 0.302	< 0.001	− 0.230	< 0.001
Waist circumference (cm)	0.349	< 0.001	0.287	< 0.001
Body mass index (kg/m^2^)	0.310	< 0.001	0.272	< 0.001
Systolic blood pressure (mmHg)	0.172	< 0.001	0.165	< 0.001
Diastolic blood pressure (mmHg)	0.190	< 0.001	0.174	< 0.001
Fasting plasma glucose (mmol/L)	0.375	< 0.001	0.323	< 0.001
Total cholesterol (mmol/L)	0.251	< 0.001	0.273	< 0.001
Triglyceride (mmol/L)	0.935	< 0.001	0.931	< 0.001
HDL-C (mmol/L)	− 0.386	< 0.001	− 0.412	< 0.001
LDL-C (mmol/L)	− 0.038	0.017	0.066	< 0.001
Vitamin D (nmol/L)	− 0.040	0.011	− 0.017	0.212
Creatinine (μmol/L)	0.091	< 0.001	0.031	0.023
Aspartate aminotransferase (IU/L)	0.094	< 0.001	0.080	< 0.001
Alanine aminotransferase (IU/L)	0.165	< 0.001	0.177	< 0.001

*ALM* appendicular lean mass; *HDL-C* high density lipoprotein cholesterol; *LDL-C* low density lipoprotein cholesterol.

### Risk of low muscle mass according to the quartiles of the TyG index

Table 4 presents the risk of low muscle mass in each quartile of the TyG index. In the unadjusted model, compared to the lowest quartile of TyG index, there was an increase in the risk of low muscle mass with an increase in the TyG index quartile with an OR (95% CI) of 1.75 (1.26–2.43), 2.35 (1.72–3.22), and 2.84 (2.09–3.86) for the second, third, and fourth quartile, respectively. The relationship was consistent even after adjusting for age, sex, prevalence of current smoking, heavy alcohol consumption, physical activity, systolic BP, creatinine, AST, ALT, TC, and vitamin D. After adjusting for confounders, the OR for low muscle mass was 1.13 (95% CI 1.07–1.20) for 1SD increase in TyG index.

**Table 4:** Multivariate logistic regression analysis for the risk of low muscle mass according to the quartiles of the TyG index.

TyG index	Q1	Q2	Q3	Q4	P-value
Unadjusted	1	1.75 (1.26–2.43)	2.35 (1.72–3.22)	2.84 (2.09–3.86)	< 0.001
Adjusted					
Model 1	1	1.62 (1.16–2.25)	2.17 (1.58–2.98)	2.76 (2.02–3.77)	< 0.001
Model 2	1	1.60 (1.15–2.24)	2.16 (1.57–2.97)	2.73 (2.00–3.74)	< 0.001
Model 3	1	1.46 (1.04–2.04)	1.78 (1.28–2.47)	2.08 (1.50–2.89)	< 0.001

Model 1: age and sex.

Model 2: age, sex, current smoker, heavy alcohol consumption, and regular exercise.

Model 3: age, sex, current smoker, heavy alcohol consumption, regular exercise, systolic blood pressure, creatinine, aspartate aminotransferase, alanine aminotransferase, total cholesterol, and vitamin D.

## Discussion

This study provides the first demonstration that increased TyG index had a significant association with low muscle mass. According to the quartiles of the TyG index, the risk of low muscle mass showed a stepwise increase. The participants with the highest quartile of the TyG index were two times more likely to have a risk of low muscle mass than those with the lowest quartile after adjusting for age, sex, lifestyle parameters, and other risk factors. A 1-SD increase in the TyG index increased the risk of low muscle mass by 13%. Therefore, elevated TyG index is associated with a risk of low muscle mass in the Korean population.

Given the increasing prevalence of sarcopenia, it is important to detect subjects with decreased muscle mass in advance. Physical inactivity, insulin resistance, oxidative stress, chronic inflammation, excess energy intake, and chronic diseases are risk factors for loss of muscle mass^22^. Among these, insulin resistance is the pivotal pathophysiological mechanism. Moon et al.^23^ suggested that weight-adjusted appendicular skeletal muscle mass (ASM) had an independent correlation with insulin resistance, metabolic syndrome, and diabetes in normal weight Korean population. We also found that subjects with T2DM have increased risk of sarcopenia after adjusting for other confounders in the Korean population^4^. Aging, the primary cause of the sarcopenia, is also related to the insulin resistance^24^. Morais et al.^25^ suggested older subjects had a higher rate of glucose disposal (*r* = 0.671, *p* < 0.001) and lower body protein metabolism than younger subjects (*p* < 0.001). Otherwise, insulin-induced activation of mTORC1 (mammalian target of rapamycin complex 1) stimulates protein synthesis. Insulin is the principal hormone with an anabolic effect, insulin resistance mediates protein breakdown and impairs muscle synthesis^26^. Skeletal muscle is the large insulin-sensitive tissue which accounts for nearly 40% of the body weight, their loss can cause insulin resistance^9,16^. Insulin resistance is the pathophysiologic mechanism of loss of skeletal muscle mass and vice versa; therefore, the TyG index may reflect muscle mass.

The golden standard method to quantify insulin secretion and resistance is the hyperinsulinemic-euglycemic clamp test^27^. Due to the invasiveness and complexity required in its clinical application, the HOMA-IR index was developed and has been validated to evaluate insulin resistance^28^. From the study using KNHANES III data, sarcopenia was associated with HOMA-IR regardless of obesity^23^. However, due to the lack of standardized insulin assays and laboratory facilities in local clinics, the routine measurement of insulin level is not recommended^29^. Moreover, there is less correlation between insulin sensitivity and HOMA-IR in lean individuals with decreased insulin secretion, HOMA-IR has limited validity in Asian populations^30^. Recently, the TyG index was introduced as a surrogate marker of insulin resistance^10^, which correlates with the hyperinsulinemic-euglycemic clamp test and HOMA-IR^11,12^. Insulin facilitates peripheral glucose uptake and hepatic glucose utilization, insulin resistance induces plasma glucose elevation and converts excess glucose to triglyceride in the liver^9,31^. In addition, insulin resistance causes an increase in the production of free fatty acids that are converted to hepatic triglyceride^32^. Therefore, the TyG index is a surrogate of insulin resistance in both peripheral and hepatic tissues. Several studies reported that the TyG index is superior to the HOMA-IR in insulin resistance-related cardiometabolic diseases such including metabolic syndrome^13^, T2DM^14^, and NAFLD^15^. From the European cohort, the predictive power of the TyG index in T2DM was stronger than fasting plasma glucose or triglyceride^33^. Furthermore, Brahimaj et al.^34^ demonstrated that TyG was a powerful predictor for T2DM among women in a 6.5-year follow-up. A study including 4986 Korean participants, the highest quartile of the TyG index group showed 2.94 times higher risk of NAFLD than the lowest quartile group^15^. Taking into consideration of the close relationship between sarcopenia and cardiometabolic diseases, the TyG index might be useful as an index of muscle mass status. The present study, we demonstrated a higher TyG index is associated with lower muscle mass using nationally representative data. Given the pathophysiological relationship between insulin resistance and low muscle mass, a positive relationship between the TyG index and sarcopenia is plausible.

To evaluate ALM, DXA is the most relevant method. However, its wide clinical application is limited by cost and availability. Therefore, there have been several attempts to predict sarcopenia using other indices including biomarkers, cytokines, hormones, and imaging techniques^35,36^. Bian et al.^37^ showed the elderly sarcopenic subjects had higher levels of IL-6 and TNF-α than control subjects. Bano et al.^38^ suggested subjects with sarcopenia had a higher level of C-reactive protein (CRP) (standardized mean difference: 0.51, 95% CI 0.26–0.77). In addition, insulin-like growth factor (IGF-1) is one of the pivotal hormones that regulates muscle growth with an endocrine/paracrine manner, stimulating muscle mass growth and decreasing proinflammatory actions. In a study including 6276 elderly subjects, IGF-1 reduction is independently associated with a decrease in skeletal muscle mass^39^. The roles of IL-6, TNF- α, CRP, and IGF-1 in insulin resistance have been widely studied^35,40^. Moreover, thigh muscle mass measured by computed tomography had inverse correlation with insulin resistance and incident T2DM over a 10-year follow-up^41^. However, these methods have not been widely used in clinical settings and require additional costs. Therefore, the TyG index, a validated index for insulin resistance which is calculated using routine laboratory measurements, may be applied as a simple and clinically useful clue for sarcopenia. Furthermore, a wide range of previous studies about insulin resistance, cardiometabolic disorders, and TyG index may support the function of TyG as a pathogenic factor for sarcopenia.

ASM is related to body size. Several methods to adjust body size are suggested, including adjustment by height squared (ASM/height^2^), weight (ASM/weight), or BMI (ASM/BMI). Despite several major consensus definitions, there are no definite methods to define skeletal muscle mass. Therefore, the prevalence of sarcopenia range from 9.9 to 40.4% as per the definition used^42^. The ASM/height^2^ index has been validated and widely adopted as an index of skeletal muscle mass. However, ASM/height^2^ may classify underweight subjects as having sarcopenia and underestimate sarcopenia in overweight and obese subjects^43^. The weight-adjusted skeletal muscle defined sarcopenic obesity had better correlation with metabolic syndrome than that defined with height-adjusted muscle mass index^44^. Considering the close relationship between the TyG index and cardiometabolic diseases, the weight-adjusted ALM index might be more appropriate to prove the interaction between the TyG index and low muscle mass. As reported in previous studies, metabolic parameters and poor lifestyle habits were positively correlated with the TyG index. Further, there was a significant association between the increased TyG index and low muscle mass.

Several studies have shown that vitamin D level is associated with an increased risk of insulin resistance and T2DM^45^. Vitamin D stimulates the differentiation and proliferation of skeletal muscle fibers, which is related to muscle mass and function^46^. Elevated vitamin D levels increase the expression of insulin receptors and lean muscle mass^47^. Epidemiological studies support the association between sarcopenia and vitamin D deficiency in elderly subjects^46^. In the 5-year longitudinal study, Granic et al.^48^ reported that the lower vitamin D level had a relationship with a faster decline in muscle strength in subjects aged > 85 years. Our study also suggested that participants with low muscle mass have decreased circulating vitamin D levels compared to those with normal muscle mass. Even after adjustment for confounding factors, including vitamin D, there was a stepwise increase in the OR of low muscle mass with an increase in the TyG index quartile. Our group, as well as others, have previously reported a relationship between sarcopenia and NAFLD^6,49^. NAFLD has been considered as a manifestation of metabolic syndrome and insulin resistance is the main pathophysiology. The skeletal muscle is the main organ for glucose disposal, and insulin resistance is known as a pivotal risk factor for both NAFLD and sarcopenia^50^. Although the present study did not have direct NAFLD data, the association between the TyG index and low muscle mass remained consistent after adjusting for aminotransferase levels.

This study has limitations. First, this study is a cross-sectional study; we could not derive a causal relationship. Second, muscle strength and physical performance were not considered because of the limited data from the KNHANES data. Third, we studied only the Asian population. Triglyceride levels differ with ethnicity^51^, and Asians have increased insulin resistance even with the same body composition. However, this study has several strengths. First, we used nationally representative data collected by the Korean government using a standard data collection method and studied many confounding factors that support the validity of this study. Second, we used DXA to measure lean mass, which is the standard method to define low muscle mass. Third, we excluded participants with confounding diseases and those on anti-hyperglycemic and lipid-lowering medications to eliminate the possible secondary causes of sarcopenia. Fourth, this is the first study to demonstrate that the TyG index could be an independent marker of low muscle mass in the general population.

In this study, an increased TyG index is related to a low muscle mass irrespective of age, sex, and other risk factors. A prospective longitudinal study in the elderly is required to understand the underlying mechanisms and causality.

## Supplementary Information

Supplementary Information.

## Data Availability

The data of the KNHANES are publicly available through the KNHANES website (https://knhanes.cdc.go.kr/).
