# Supplementary material for: Association of the triglyceride and glucose index with low muscle mass: KNHANES 2008–2011
Source: Sci Rep. 2021 Jan 11;11:450. doi: 10.1038/s41598-020-80305-1 (PMC7801612; doi:10.1038/s41598-020-80305-1)
Supplement: Supplementary file 1 — Supplementary Information. [file 41598_2020_80305_MOESM1_ESM.pdf]

Original article

Association of the triglyceride and glucose index with low muscle mass: KNHANES 2008-2011

Jung A Kim[1], Soon Young Hwang[2], Ji Hee Yu,[1] Eun Roh[1], So-hyeon Hong[1], You-Bin Lee[1], Nam Hoon Kim[1], Hye Jin Yoo[1], Ji A Seo[1], Nan Hee Kim[1], Sin Gon Kim[1], Sei Hyun Baik[1], and Kyung Mook Choi[1*]

[1]Division of Endocrinology and Metabolism, Department of Internal Medicine, College of Medicine, Korea University, Seoul, Republic of Korea

[2]Department of Biostatistics, Korea University College of Medicine, Seoul, Republic of Korea

[*]Corresponding. medica7@gmail.com

Supplementary Table 1. Baseline characteristics according to the quartiles of the TyG index in men and women

	Men					Women				
	TyG index					TyG index				
	Q1	Q2	Q3	Q4	P-value	Q1	Q2	Q3	Q4	P-value
n	759	899	1,054	1,286		1,606	1,478	1,312	1,083	
Age (years)	58.8±11.9	57.9±11.7	57.0±11.4	55.4±10.8	<0.001	52.4±10.4	56.2±11.3	58.3±11.3	60.5±11.1	<0.001
Waist circumference (cm)	79.8±8.5	82.6±8.7	85.0±7.6	87.7±7.0	<0.001	76.3±8.3	79.3±8.7	81.6±8.9	84.1±8.6	<0.001
Body mass index (kg/m^2)	22.2±2.8	23.2±3.0	23.9±2.7	24.8±2.6	<0.001	22.6±2.8	23.6±3.0	24.3±3.3	24.9±3.1	<0.001
Systolic blood pressure (mmHg)	121.1±17.4	123.4±16.9	124.5±17.1	126.9±16.2	<0.001	115.9±17.3	120.9±17.5	123.8±17.6	129.0±18.3	<0.001
Diastolic blood pressure (mmHg)	77.4±10.6	78.9±10.5	80.7±10.6	82.9±10.4	<0.001	74.3±10.0	76.4±9.9	77.6±10.1	79.6±10.4	<0.001
Fasting plasma glucose (mmol/L)	5.0±0.5	5.2±0.5	5.3±0.5	5.6±0.6	<0.001	5.0±0.4	5.1±0.5	5.3±0.5	5.4±0.5	<0.001
Total cholesterol (mmol/L)	4.5±0.8	4.8±0.8	5.0±0.8	5.2±1.0	<0.001	4.8±0.8	5.0±0.8	5.3±0.9	5.5±0.9	<0.001
Triglyceride (mmol/L)	0.7±0.1	1.0±0.1	1.5±0.2	2.6±0.7	<0.001	0.7±0.1	1.1±0.1	1.5±0.2	2.5±0.6	<0.001
HDL-C (mmol/L)	1.4±0.3	1.3±0.3	1.2±0.3	1.1±0.2	<0.001	1.4±0.3	1.3±0.3	1.2±0.2	1.1±0.2	<0.001
LDL-C (mmol/L)	2.9±0.7	3.0±0.8	3.1±0.8	2.9±0.9	<0.001	3.0±0.7	3.2±0.8	3.4±0.8	3.2±0.9	<0.001
Aspartate aminotransferase (IU/L)	24.4±11.8	23.8±11.8	24.7±18.0	27.2±17.1	<0.001	20.2±7.3	21.0±7.6	21.3±8.2	23.0±9.3	<0.001
Alanine aminotransferase (IU/L)	20.5±12.8	21.6±12.9	24.8±29.0	29.0±17.5	<0.001	15.9±9.1	17.5±9.9	18.7±12.3	21.7±16.2	<0.001
Blood urea nitrogen (mmol/L)	6.0±1.5	5.6±1.5	5.5±1.5	5.3±1.4	<0.001	5.2±1.5	5.2±1.5	5.1±1.4	5.2±1.4	0.325
Creatinine (μmol/L)	79.6±8.8	88.4±17.7	88.4±8.8	88.4±8.8	<0.001	61.9±8.8	61.9±8.8	61.9±8.8	61.9±8.8	<0.001
Vitamin D (nmol/L)	21.6±7.6	21.3±7.3	21.2±7.2	20.7±7.1	<0.001	17.7±6.7	18.1±6.9	17.9±6.7	18.2±6.9	0.143
TyG index	7.9±0.2	8.4±0.1	8.7±0.1	9.3±0.3	0.047	7.8±0.2	8.4±0.1	8.7±0.1	9.3±0.2	<0.001
ALM/weight (%)	35.2±2.9	34.2±2.8	33.8±2.7	33.0±2.5	<0.001	27.6±2.6	26.7±2.6	26.3±2.6	25.9±2.4	<0.001
Low muscle mass (%)	13(1.7%)	21(2.3%)	33(3.1%)	61(4.7%)	<0.001	45(2.8%)	79(5.3%)	99(7.5%)	97(9.0%)	<0.001
Hypertension (%)	251(33.1%)	323(36.1%)	441(42.2%)	625(48.7%)	<0.001	321(20.1%)	449(30.5%)	534(40.8%)	563(52.2%)	<0.001
Current smoker (%)	252(33.4%)	317(35.5%)	412(39.4%)	547(42.6%)	<0.001	51(3.2%)	57(3.9%)	55(4.2%)	83(7.7%)	<0.001

Heavy alcohol consumption (%)	194(25.7%)	233(26.1%)	339(32.3%)	530(41.5%)	<0.001	75(4.7%)	92(6.3%)	69(5.3%)	79(7.4%)	0.024
Regular exercise (%)	225(29.8%)	259(29.0%)	278(26.5%)	304(23.8%)	<0.001	408(25.7%)	358(24.4%)	329(25.3%)	233(21.6%)	0.094

Data are expressed as mean ± standard deviation or n (%).

ALM, Appendicular lean mass; HDL-C, high-density lipoprotein cholesterol; LDL-C, low-density lipoprotein cholesterol; TyG index, triglyceride-glucose index
